# Meteorological Influences on the Incidence of Aneurysmal Subarachnoid Hemorrhage – A Single Center Study of 511 Patients

**DOI:** 10.1371/journal.pone.0081621

**Published:** 2013-12-02

**Authors:** Marian Christoph Neidert, Michael Sprenger, Heini Wernli, Jan-Karl Burkhardt, Niklaus Krayenbühl, Oliver Bozinov, Luca Regli, Christoph Michael Woernle

**Affiliations:** 1 University Hospital Zurich, Department of Neurosurgery, Zurich, Switzerland; 2 Institute for Atmospheric and Climate Science, ETH Zurich, Zurich, Switzerland; University Hospital Zurich, France

## Abstract

**Objective:**

To assess the potential meteorological influence on the incidence of aneurysmal subarachnoid hemorrhage (SAH). Previous studies used inhomogeneous patient groups, insufficient study periods or inappropriate statistics.

**Patients and Methods:**

We analyzed 511 SAH admissions between 2004 and 2012 for which aneurysmal rupture occurred within the Zurich region. The hourly meteorological parameters considered are: surface pressure, 2-m temperature, relative humidity and wind gusts, sunshine, and precipitation. For all parameters we investigate three complementary statistical measures: i) the time evolution from 5 days before to 5 days after the SAH occurrence; ii) the deviation from the 10-year monthly mean; and iii) the change relative to the parameter's value two days before SAH occurrence. The statistical significance of the results is determined using a Monte Carlo simulation combined with a re-sampling technique (1000×).

**Results:**

Regarding the meteorological parameters considered, no statistically significant signal could be found. The distributions of deviations relative to the climatology and of the changes during the two days prior to SAH events agree with the distributions for the randomly chosen days. The analysis was repeated separately for winter and summer to exclude compensating effects between the seasons.

**Conclusion:**

By using high-quality meteorological data analyzed with a sophisticated and robust statistical method no clearly identifiable meteorological influence for the SAH events considered can be found. Further studies on the influence of the investigated parameters on SAH incidence seem redundant.

## Introduction

Spontaneous subarachnoid hemorrhage (SAH) accounts for about 5% of all strokes and it affects approximately 30,000 people in the United States per year.[1], [2] With an overall mortality of around 45%, SAH is a major contributor to the stroke-related loss of productive life years, since it occurs at younger ages than ischemic stroke or intracerebral hemorrhage.[3]–[5] Therefore, much research effort aims at evaluating risk factors for SAH. The risk factors female gender, age, arterial hypertension and tobacco use are supported by strong data[6]–[8] and in addition, genetic predisposition, drug and alcohol abuse are the most commonly discussed ones.[9]–[12] Previous research on the meteorological influence on the incidence and pathophysiology of aneurysmal SAH has been triggered by the clinical observation that SAH occurs in clusters, apparently more frequently in a particular season. Several studies have linked SAH occurrence to specific seasonal influences and weather pattern[13]–[22], whereas other studies failed to show any seasonal or meteorological association[23]–[30]. To assess the influence of weather on SAH in the region of Zurich, we analyzed hourly meteorological parameters from three measurement sites around Zurich gathered by the Swiss National Weather Service (MeteoSwiss). The strengths of our study are first, a homogeneous patient group of 511 patients that suffered from intracranial aneurysm rupture within a locally well-confined region, and second, high-quality meteorological data analyzed with a sophisticated and robust statistical method.

Thus, the meteorological influences are expected to be less diluted than in series that lump together meteorological data from various climate zones and the meteorological data provided are very detailed and reliable.

## Patients and Methods

### Patients

Between January 2004 and March 2012 we retrospectively analyzed the medical records of 511 consecutive patients admitted to our department for spontaneous SAH due to rupture of an intracranial aneurysm. The day of symptom onset (not the admission day) was considered for the analysis. Patients were included only if they had clinical and neuroradiological (computed tomography) findings consistent with SAH as well as angiographically confirmed aneurysm as the source of bleeding. In addition, patients were excluded if the bleeding occurred outside the catchment area of the meteorological data (state of Zurich) or if the time point of aneurysmal rupture was unclear. Age, gender distribution, location of the aneurysm, and severity grades (initial Glasgow Coma Scale, Hunt and Hess Grade, Fisher Grade, and World Federation of Neurosurgical Societies Grade) were evaluated.

### Ethics statement

Acquisition of patient data was performed by physicians directly involved in the treatment or follow-up of the involved subjects, further analyses were based on anonymous data. In accordance with institutional guidelines and federal law, neither an ethics approval nor written informed consent was obtained (waiver issued by Cantonal Ethics Committee, KEK Zürich).

### Target area

The state of Zurich, Switzerland, has about 1.4 million inhabitants and covers an area of 1729 km^2^ (population density: 813/km^2^). The state consists mostly of shallow river valleys that drain towards the Rhine (north) and mountains in the northwest and southeast; the highest point is at 1291 m (Schnebelhorn, mountain) and the lowest point at 330 m (at the northern border towards the Rhine).

### Meteorological data

All meteorological data are taken from the operational measurement network of the Swiss National Weather Service (MeteoSwiss). The following fields are considered: temperature (in °C), relative humidity (in %), and wind gusts (in m/s) at 2 m above ground, surface pressure (in hPa), precipitation (in mm/h), and sunshine duration (in min per hour). All fields are available in hourly intervals and we restricted the analysis to three stations covering the target area: Hörnli (HOE, 8° 56′ 29″E/47° 22′ 14″N/1144 m a.s.l.), Zurich (SMA, 8° 33′ 57″E/47° 22′ 41″N/556 m a.s.l.), and Wädenswil (WAE, 8° 40′ 36″E/47° 13′ 16″N/463 m a.s.l.). Further information on the measurement sites and on the quality of the data can be obtained on www.meteoswiss.ch.

### Statistical Analysis

The statistical analysis and the illustration of the data are done using Matlab (www.mathworks.com), and performed in several distinct steps. First, each SAH day is characterized by a single daily value for each meteorological parameter. For temperature, surface pressure and relative humidity this is the daily mean; the maximum is taken for winds gusts, the daily sum for precipitation and, finally, for sunshine the integrated minutes with sunshine between 10 and 15 UTC. This time restriction takes into account the seasonal shift in sunrise and sunset, and guarantees a measure that is comparable throughout the year.

**Figure 1:**
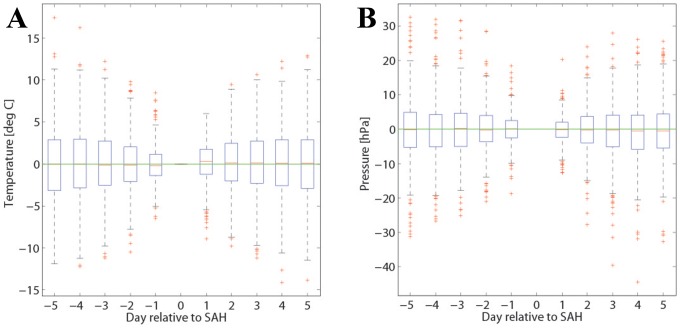
Time evolution from 5 days before to 5 days after SAH occurrence. The statistical analysis for measurement site SMA and for temperature (A) and pressure (B) is depicted.

In a first approach, we then determine the same daily metric for the preceding and subsequent five days, and only consider the difference relative to the SAH day. This analysis indicates whether systematic temporal changes occur during the days when SAH events occur. An example is shown in Fig. 1A for temperature at the station SMA. Day 0 corresponds to the SAH events and the box plots for the preceding and subsequent days show the distribution of the mean temperatures relative to the temperature at the SAH day. In this example we see no systematic temperature evolution during the days preceding and following SAH events.

**Figure 2:**
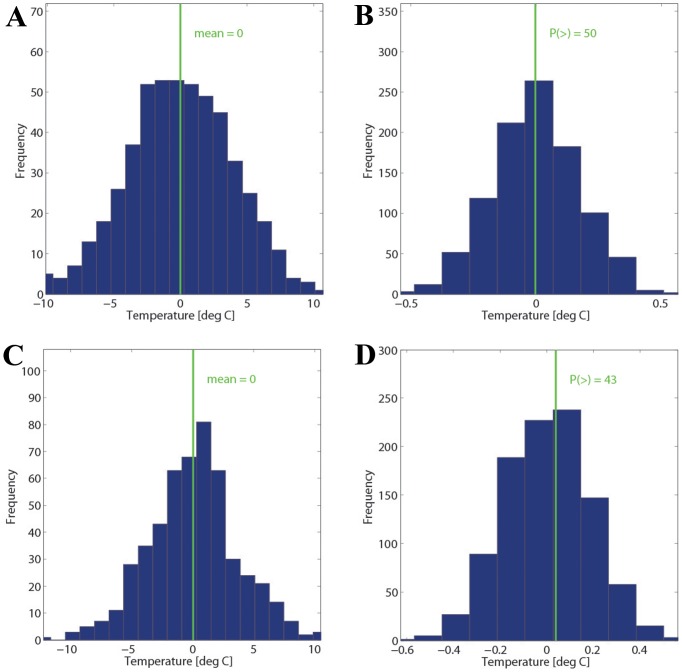
Temperature deviation from the 10-year monthly mean and temperature changes relative to two days before SAH occurrence. Panel (A) shows a histogram of observed temperatures on SAH days relative to the 10-year monthly means. The green value gives the mean over all observed temperature anomalies on SAH days; (B) the green line corresponds to the observed mean given in panel (A) and the blue distribution results from a Monte Carlo simulation with 1000 members, assuming that no relationship between SAH and temperature exists. Panels (C) and (D) show correspondingly the short-term change in temperature relative to the SAH day. Hence, (C) shows the distribution of the observed temperature changes from 2 days before to the SAH day (see text for details) - in the mean, no temperature change is discernible (green line); (D) shows the observed mean from panel (C) as a green line and the distribution from the Monte Carlo simulations. The P-value gives the number of Monte Carlo members to the right of the observed value.

A second, complementary approach starts again with the definition of a daily characteristic for each SAH day and each meteorological parameter. For instance, for each SAH day we calculate the deviation of the daily mean temperature to the 10-year climatological monthly mean and plot their distribution, as shown in Fig. 2A. Then, the mean of this distribution is determined, shown as the green line in Fig. 2A. The distribution average of −0.002°C indicates that the temperature at SAH days is essentially what would be expected from a monthly climatology, i.e., they are not “unusual” in terms of meteorological conditions.

To assess the statistical significance of the distribution averaged deviation, we apply a Monte Carlo simulation. We repeat the whole analysis illustrated in Fig. 2A, leading to a distribution mean temperature, but not for the observed SAH days but for a random selection of 511 days during the years 2005 – 2011 (adjusted time period in order to ensure whole-year datasets). The 511 randomly selected days are such that we keep the same monthly frequencies as in the observed sample. Again a distribution of mean temperature values results, although this one is due to the randomization not related to SAH. We repeat this random analysis 1000 times (re-sampling method), resulting in a distribution of 1000 mean temperatures shown in Fig. 2B (referred to as “random mean distribution” in the following). The temperature mean during the observed SAH days can then be compared to the distribution of the 1000 randomly determined temperature means. If the SAH temperature mean lies outside of the 5% or 95% percentile of the random mean distribution, a statistically significant effect has been found. On the other hand, if the observed value lies near the center of the distribution, no significant meteorological influence can be inferred, as is the case in Fig. 2B. When plotting, the median of the random mean distribution is shifted to 0 and such is the mean for the SAH days, which in this case is very close to the center of the random mean distribution.

Finally, the same method can be used to statistically establish if a weather shift, e.g., a rapid change of temperature, goes along with the SAH days. Basically, the steps are as before, except for the definition of the daily metric. Before, we compared the temperature to a monthly climatology, and thus established if there is a climatologically significant signal associated with SAH events. Now we take the temperature difference between the SAH day and two days before as the daily metric. If one or both of the preceding days were also SAH days, we shift the preceding days accordingly, so that we compare the SAH temperature with the temperature of a non-SAH day at least two days before. It is hoped that the restriction to non-SAH days before contributes to a sharper signal. No correction for the seasonal cycle is necessary for this kind of analysis. As an example, Fig. 2C+D show the weather shift analysis for temperature. The SAH days show no systematic temperature change (mean value close to 0) and no significant deviation from the random mean distribution.

Theoretically, the outcome of our analysis could be different for the warm and the cold season. For instance, SAH incidence might be related to warm anomalies during the warm season and to cold anomalies during the cold season, or vice versa. Such a seasonal effect would not be detected by our method. To exclude this possibility, we repeated the whole analysis separately for the cold and warm seasons (data not shown). However, no significant differences resulted, confirming that no obvious seasonal dependence is of importance.

## Results

### Clinical data

In the time period between January 2004 and March 2012 we included 511 consecutive patients that suffered from SAH due to an aneurysm rupture within the state of Zurich. Our study group consisted of 340 females (66.5%) and 171 males (33.5%; female to male ratio  = 2∶1), and the mean age was 53 years (range 15 – 97). Briefly, the ruptured aneurysm was located within the anterior circulation in 84.1% of the cases, whereas 15.9% had a ruptured aneurysm within the posterior circulation. The initial Glasgow Coma Scale (GCS) ranged from 3 to 15 and was between GCS 13–15 in 295 cases (57.7%), GCS 9–12 in 36 patients (7.0%), and GCS 3–8 in 180 (35.2%) of all included SAH admissions. The severity grades were distributed as follows: Hunt and Hess Grade (Grad 1: 39, 7.6%; Grade 2: 227, 44.4%; Grade 3: 85, 6.6%; Grade 4: 68, 13.3%; Grade 5: 92, 18.0%), Fisher Grade (Grade 1: 28, 5.5%; Grade 2: 76, 14.9; Grade 3: 181; 35.8%; Grade 4: 224; 43.8%), and World Federation of Neurosurgical Societies Grade (Grade 1: 180, 35.2%; Grade 2: 100, 19.6%; Grade 3: 30, 5.9%; Grade 4: 78, 15.3%; Grade 5: 123, 24.0%).

### Meteorological data

Three complementary statistical analyses have been performed, as outlined in the previous section: i) the time evolution from 5 days before to 5 days after the SAH occurrence; ii) the deviation from the 10-year monthly mean with re-sampling; and iii) the change relative to the parameter's value two days before SAH occurrence.

#### Time evolution of meteorological parameters

**Figure 3:**
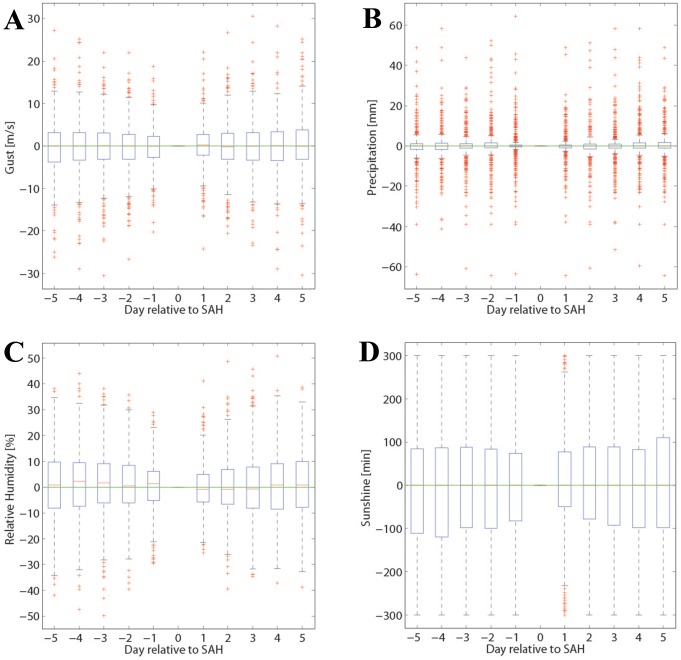
Time evolution of meteorological parameters (gust, precipitation, relative humidity and sunshine) around the SAH day, as in **Figure 1A and B**. The boxplots are for site SMA.

The results for analysis i) are summarized in Figs. 1A and 1B and Fig. 3 (A–D), which show the mean time evolution of all meteorological parameters considered during the 10 days centered at the SAH day at the measurement site SMA. No distinct systematic weather shifts are discernible (the same is true at the two other stations). For instance, if SAH events were related to the passage of a weather front we could see a marking in all parameters. But averaged temperature changes stay below 0.2°C, with a slightly lower value before than after the SAH day; relative humidity is slightly higher before than after the SAH day, but the maximum difference stays below 2%; and pressure fluctuates by less than 1 hPa during the 10-day period. Also changes in sunshine, wind gusts and precipitation exhibit no clear signal. Note that these fluctuations of the mean values are much smaller than the variability. As an example, mean temperature varies during the 10 days by about 0.2°C but the variability (Fig. 1A) amounts to about 5°C. The same applies for the other meteorological fields: the mean changes are very small compared to the event-to-event variability. This clearly indicates that we cannot identify any systematic temporal evolution of the meteorological parameters. Finally, it is worthwhile to mention that the results are very consistent for the three measurement sites (SMA, WAE, HOE) considered. The results from this analysis are also separately depicted in the Figure S1.

#### Deviation from the 10-year monthly mean with re-sampling

**Figure 4:**
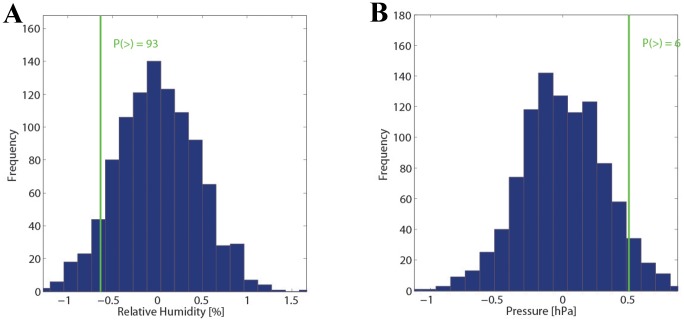
Deviation from the 10-year monthly mean for humidity and pressure. Statistical analysis as in Figure 2B for relative humidity (A) and pressure (B), corresponding to the parameters with highest statistical signal. The right panels compare the observed value (green line) with the distribution from a Monte Carlo simulation with 1000 members (see text for details).

The complete summary of results from this analysis– also shown separately for the three different stations – is depicted in Figure S2 (observed values) and Figure S3 (distribution of the Monte Carlo simulation). Figure 4 summarizes the main findings for analysis ii) by depicting the two parameters with the greatest deviation from climatology at the station SMA. Figure 4A shows the results for humidity and Fig. 4B for pressure – all other parameters are shown in the Material (Fig. S3). The mean temperature (Fig. 2B) and mean gust values (Fig. S3) for all SAH days match very well with the climatology; pressure (Fig. 4B) and sunshine (Fig. S3) are slightly higher than expected from climatology; and finally, relative humidity is slightly lower (Fig 4A). However, the observed values for all meteorological fields fall well within the distribution from the randomized samples. Hence, the deviations from climatology are not statistically significant at the 5%-level. The highest p-values result for pressure (p∼6%) and relative humidity (p∼93%), which is confirmed for the other two stations (WAE, HOE). A similar consistency between the three stations is basically also found for the other meteorological fields where also no statistical significance at the 5%-level is reached for any field. In summary, the statistical analysis indicates the following meteorological situation: the SAH days agree with climatology for mean temperature and wind gusts; the days are drier and sunnier than climatology, which is consistent with a higher pressure. However, all deviations are very small, especially if compared to the overall variability of the parameters, and not statistically significant.

#### Short-term changes with re-sampling

**Figure 5:**
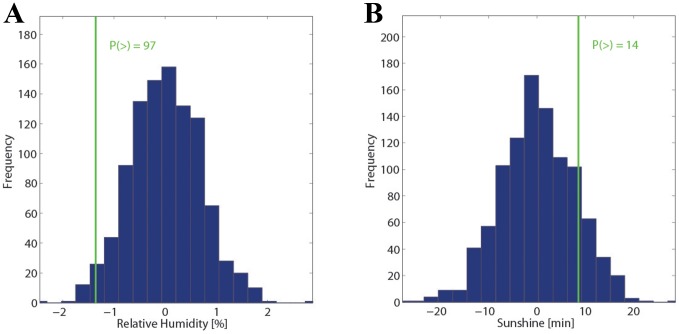
Short-time changes in humidity and sunshine. A statistical analysis as in Figure 2D for the short-time change in relative humidity (A) and sunshine (B) is depicted. The observed changes (green line) are compared to the distribution from a Monte Carlo simulation with 1000 members (see text for details).

Figure 5 shows the main results from the statistical analysis on changes in the meteorological parameters during the 2 days before the SAH day at the station SMA. The complete summary of results from this analysis– also shown separately for the three different stations – is depicted in Figure S4 (observed values) and Figure S5 (distribution of the Monte Carlo simulation). Again, the observed values remain well within the limits from the distributions, i.e., no statistically significant short-term change in the meteorological parameters is discernible before the SAH days except for relative humidity (Fig. 5A; p-value of 97%). Other p-values are 17% for wind gusts (Fig. S5), 14% for sunshine (Fig. 5B), and values close to 50% for the other parameters (Fig. S5). These values are consistent in the direction of the 2-day changes at the other two stations (HOE, WAE) and are often comparable in amplitude (not shown). Partly, they are slightly more extreme than at SMA (e.g., wind gusts at HOE: 8.4%) or less extreme (e.g., sunshine at WAE: 25.2%). In summary, the pattern would correspond to slightly gustier wind conditions, drier air and more sunshine on the SAH days compared to the immediate days before. However, note that the absolute shifts (∼0.5 m/s for gusts; ∼1% for relative humidity; and 10 min for sunshine) are very small, and their meteorological relevance is questionable and in most cases, not statistically significant.

### Seasonality

**Figure 6:**
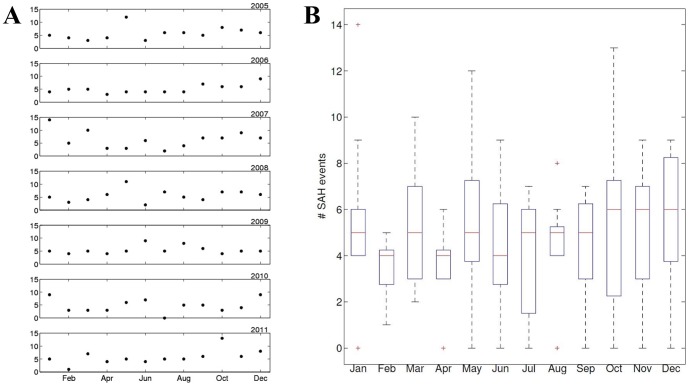
Seasonal distribution of SAH events. The seasonal distribution of SAH events between 2005 and 2011 (whole-year data sets) is depicted for individual years (A) and for the entire studied time period (B). While there are monthly peaks in individual years (A), no obvious peak can be identified when analyzing the entire studied time period (B).

When just looking at the seasonal distribution of SAH events between 2005 and 2011 (whole-year data sets) without considering meteorological data (Fig. 6), it can be seen that there are monthly peaks in individual years (Fig. 6A; i.e., May 2005, January 2007, or October 2011). However, when plotting the seasonal cycle for all years (2005–2011) in one diagram (Fig. 6B), these peaks level out and SAH events are fairly evenly distributed over the entire year.

## Discussion

All analyses performed in our study did not show a clearly identifiable influence of the meteorological parameters temperature, wind gusts, surface pressure, precipitation, relative humidity, and sunshine duration for the 511 SAH events considered. The first analysis (i), looks at the time evolution from 5 days before to 5 days after the SAH occurrence and thereby allows identifying systematic weather shifts related to SAH events. No systematic changes have been found. This analysis, however, makes no statement on how the meteorological parameters compare to the monthly climatology. For instance, it is also of interest if the temperature on SAH days deviates significantly from the monthly climatology. Therefore, we applied a second method (ii) which is particularly suitable to address this issue. It is interesting to compare the short-term weather shifts (iii) with the deviation from climatology (ii). The two signals agree for relative humidity (drier), sunshine (sunnier) and temperature; but, they are different for gusts and for pressure. No immediate pressure change is found during the days before the SAH, whereas the pressure is slightly higher than expected from the monthly climatology, i.e., some SAH days are related to a stable high pressure system. However, this pattern is not systematic since we do not find on average a strong positive deviation in surface pressure from climatology. Interesting is also the discrepancy between the wind gust analyses. The weather becomes slightly gustier towards the SAH day, but the gustiness matches with the climatological expectation. Hence, the wind gusts preceding the SAH days are weaker than expected from climatology.

Several studies have linked SAH occurrence to specific seasonal influences and weather patterns,[13]–[22] whereas other studies failed to show any seasonal or meteorological associations.[23]–[30]

Several explanations for this discrepancy of results have been discussed in the past. One reason might be the great heterogeneity of the reported data, not only considering the geographical areas and different qualities of meteorological data, but also in terms of study design. Whereas some of the more recent studies focused on a single-center such as Abe et al.,[15] Beseoglu et al.,[23] and Setzer et al.,[13] other recent studies combined retrospective data from various locations and climate zones, such as Cowperthwaite et al.[30] or McDonald et al.[31] Also, the studies time-periods differ significantly. For the previously discussed studies, the considered time period was between 12 months[15] and seven years.[31] Especially when analyzing the seasonal distribution, a sufficiently long study period is important. In our series for instance, we observed seasonal peaks for individual years, but an even distribution of SAH events over the entire time period studied. In addition, the time point of the SAH event used for statistical calculations is often ill-defined. Some authors used the time of the emergency call,[15] whereas most other authors calculated with the day of hospital admission.[30] In our experience, the onset of symptoms due to the rupture of an intracranial aneurysm might be days before admission, especially in milder cases. Therefore, the day of symptom onset was used for our calculations. Moreover, it is possible that seasonal and/or weather fluctuations do not have a direct pathophysiological impact and thereby cause aneurysmal rupture. Weather might influence SAH occurrence indirectly by inducing changes in lifestyle (physical activity, consumption of alcohol and tobacco.[30]

### Limitations of our study

We are aware of some notable limitations of our study. This study focuses on one center and a relatively small catchment area (Zurich region). However, in our opinion this represents also a major strength of our approach, since we can provide high-quality local meteorological data without mixing meteorological influences such as by pooling data from various climate zones. The data collection was performed retrospectively and the analysis is based on the day of symptom onset (not necessarily the admission day). Impacts of within-day and spatially confined meteorological fluctuations are theoretically still possible, but are beyond the scope of this study. Such an analysis would be much more complex to perform as it required detailed information about the exact time and location of SAH occurrences and more importantly, meteorological observational data on short temporal and small spatial scales are not readily available. Thus, it seems highly unlikely that future studies on the parameters considered will show a clear impact of weather on SAH incidence in case they are performed with high-quality clinical and meteorological data and carried out using an appropriate statistical method.

## Conclusion

Our study negates a clearly identifiable meteorological or seasonal influence for the 511 SAH events analyzed. Impacts of extremely short within-day and spatially confined meteorological fluctuations are theoretically possible and cannot be excluded. However, it would be virtually impossible to obtain a robust combined medical and meteorological dataset of SAH events on short temporal and small spatial time scales. Thus, future studies are extremely unlikely to show a systematic influence of the parameters considered when based on a sufficient study period, on high-quality data, and on robust statistics.

## Supporting Information

Figure S1 **Time evolution of the meteorological parameters gust, surface pressure, precipitation, relative humidity, sunshine duration, and mean temperature around the SAH day is depicted.** The same daily metric for the preceding and subsequent five days are shown as box plots for the three stations SMA, WAE, and HOE separately.
(PDF)

Figure S2 **Histogram of observed parameters (gust, surface pressure, precipitation, relative humidity, sunshine duration, and mean temperature) on SAH days relative to the 10-year monthly means.** The green value gives the mean over all observed temperature anomalies on SAH days. The values are shown for the three stations SMA, WAE, and HOE separately.
(PDF)

Figure S3 **Histogram of the mean deviation from the 10-year monthly means from a Monte Carlo simulation (random selection of 511 days within the same time period) with 1000 re-samplings, assuming that no relationship between SAH and temperature exists.** The green line corresponds to the observed mean given in panel Figure S2 and the blue distribution results from the Monte Carlo simulation. The P-value gives the number of Monte Carlo member to the right of the observed value.
(PDF)

Figure S4 **Histogram of observed parameters (gust, surface pressure, precipitation, relative humidity, sunshine duration, and mean temperature) on SAH days relative to 2 days prior to the bleeding event (short-time change).** The green value gives the mean over all observed temperature anomalies on SAH days. The values are shown for the three stations SMA, WAE, and HOE separately.
(PDF)

Figure S5 **Histogram of the mean deviation from 2 days prior to the bleeding event from a Monte Carlo simulation (random selection of 511 days within the same time period) with 1000 re-samplings, assuming that no relationship between SAH and temperature exists.** The green line corresponds to the observed mean given in Figure S4 and the blue distribution results from the Monte Carlo simulation. The P-value gives the number of Monte Carlo member to the right of the observed value.
(PDF)
