# Supplementary material for: Meteorological Influences on the Incidence of Aneurysmal Subarachnoid Hemorrhage – A Single Center Study of 511 Patients
Source: PLoS One. 2013 Dec 2;8(12):e81621. doi: 10.1371/journal.pone.0081621 (PMC3847045; doi:10.1371/journal.pone.0081621)

Supplemental Figure S1

Time evolution of the meteorological parameters gust, surface pressure, precipitation, relative humidity, sunshine duration, and mean temperature around the SAH day is depicted. The same daily metric for the preceding and subsequent five days are shown as box plots for the three stations SMA, WAE, and HOE separately.

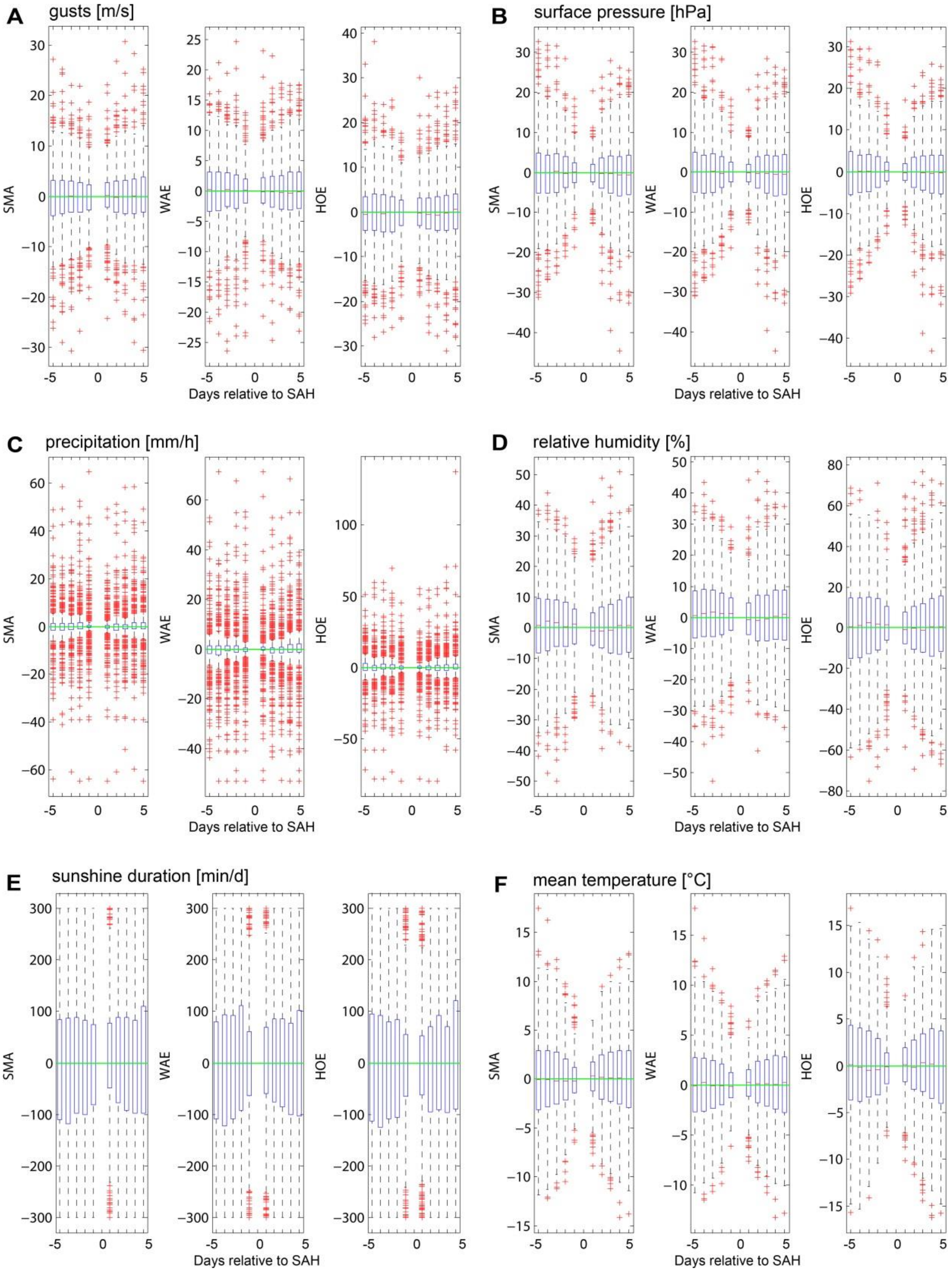

Supplement: Figure S1 — Time evolution of the meteorological parameters gust, surface pressure, precipitation, relative humidity, sunshine duration, and mean temperature around the SAH day is depicted. The same daily metric for the preceding and subsequent five days are shown as box plots for the three stations SMA, WAE, and HOE separately. (PDF) [file pone.0081621.s001.pdf]
