# Supplementary material for: Meteorological Influences on the Incidence of Aneurysmal Subarachnoid Hemorrhage – A Single Center Study of 511 Patients
Source: PLoS One. 2013 Dec 2;8(12):e81621. doi: 10.1371/journal.pone.0081621 (PMC3847045; doi:10.1371/journal.pone.0081621)

Supplemental Figure S2

Histogram of observed parameters (gust, surface pressure, precipitation, relative humidity, sunshine duration, and mean temperature) on SAH days relative to the 10-year monthly means. The green value gives the mean over all observed temperature anomalies on SAH days. The values are shown for the three stations SMA, WAE, and HOE separately.

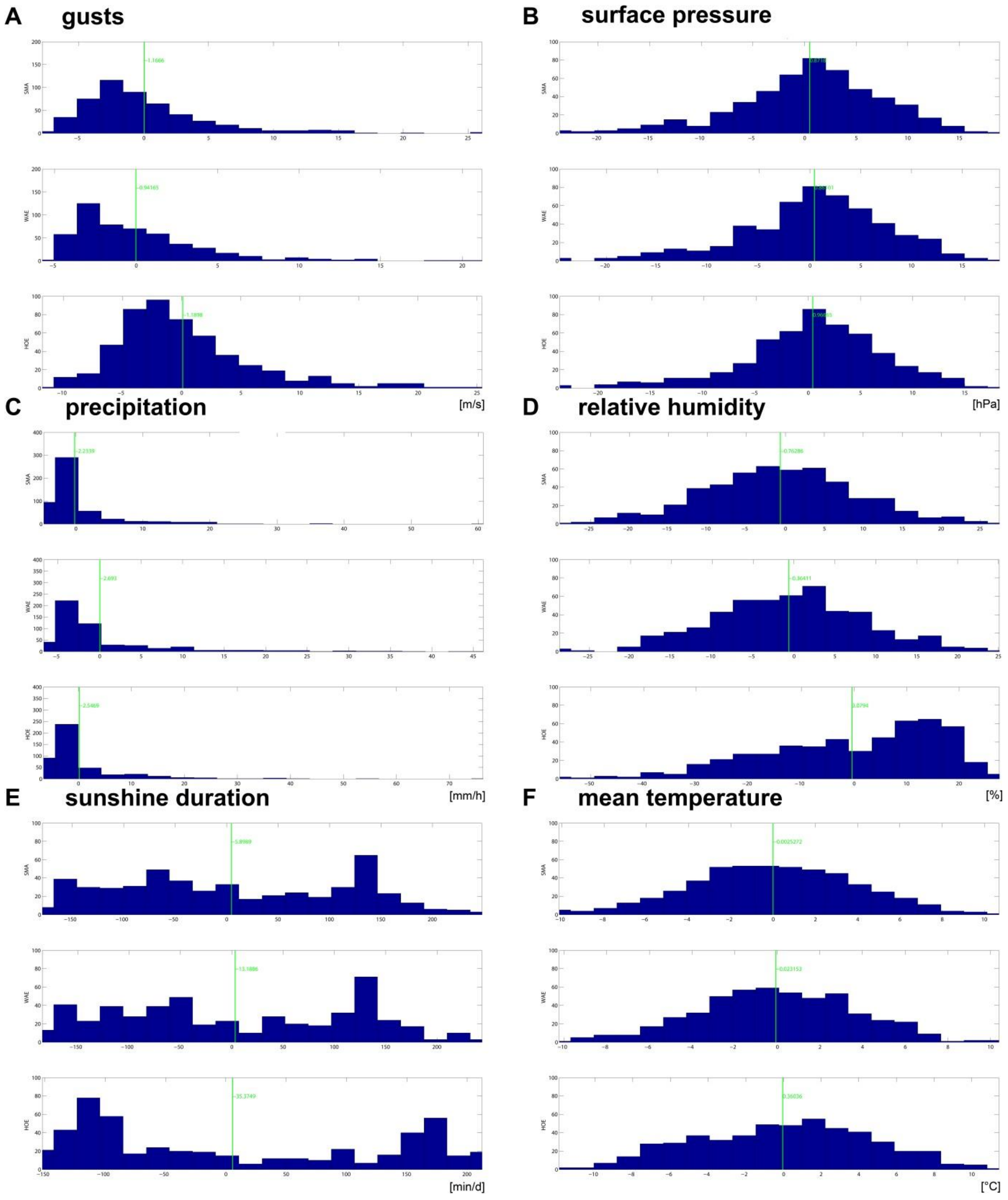

Supplement: Figure S2 — Histogram of observed parameters (gust, surface pressure, precipitation, relative humidity, sunshine duration, and mean temperature) on SAH days relative to the 10-year monthly means. The green value gives the mean over all observed temperature anomalies on SAH days. The values are shown for the three stations SMA, WAE, and HOE separately. (PDF) [file pone.0081621.s002.pdf]
