# Supplementary material for: Meteorological Influences on the Incidence of Aneurysmal Subarachnoid Hemorrhage – A Single Center Study of 511 Patients
Source: PLoS One. 2013 Dec 2;8(12):e81621. doi: 10.1371/journal.pone.0081621 (PMC3847045; doi:10.1371/journal.pone.0081621)

Supplemental Figure S4

Histogram of observed parameters (gust, surface pressure, precipitation, relative humidity, sunshine duration, and mean temperature) on SAH days relative to 2 days prior to the bleeding event (short-time change). The green value gives the mean over all observed temperature anomalies on SAH days. The values are shown for the three stations SMA, WAE, and HOE separately.

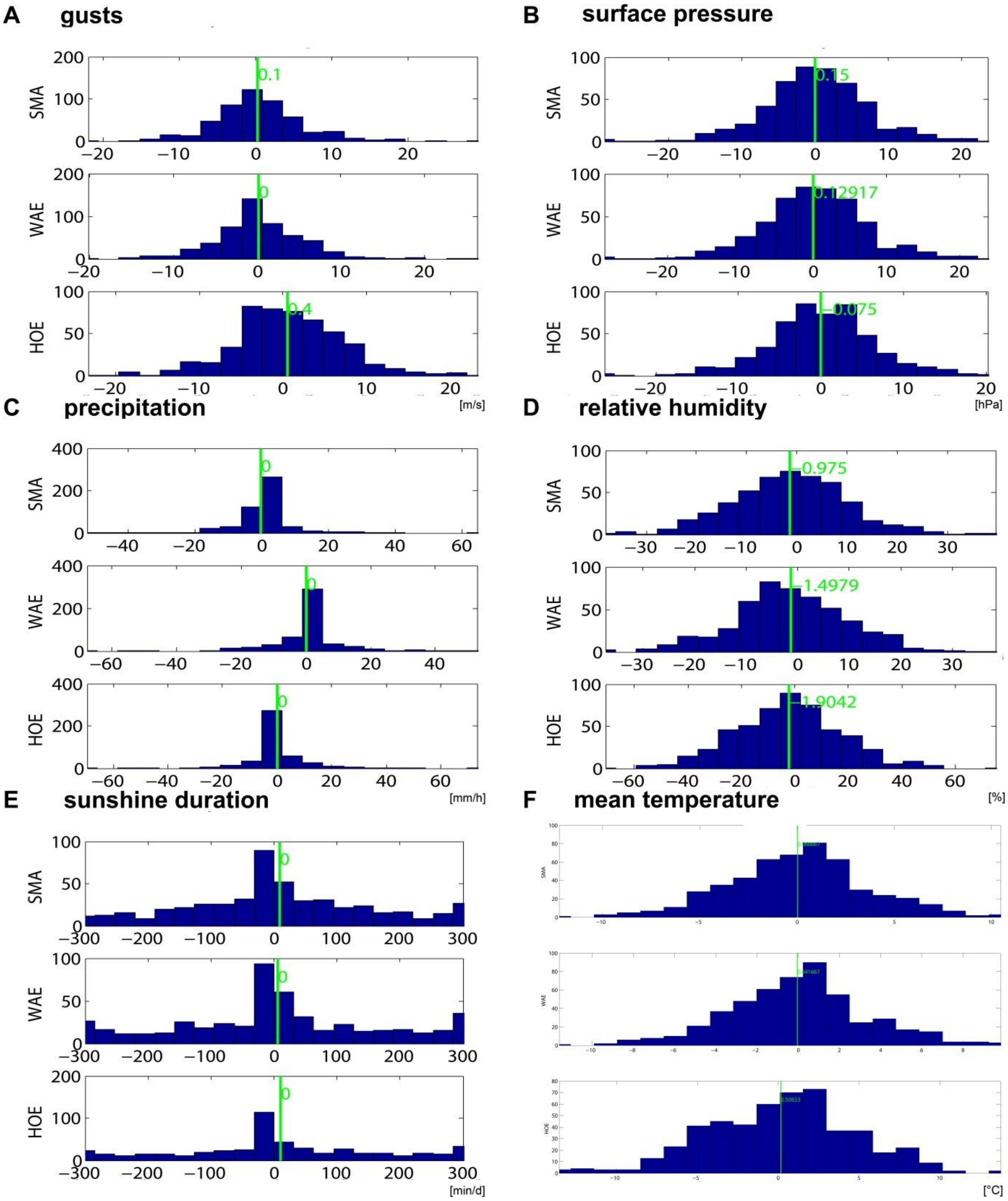

Supplement: Figure S4 — Histogram of observed parameters (gust, surface pressure, precipitation, relative humidity, sunshine duration, and mean temperature) on SAH days relative to 2 days prior to the bleeding event (short-time change). The green value gives the mean over all observed temperature anomalies on SAH days. The values are shown for the three stations SMA, WAE, and HOE separately. (PDF) [file pone.0081621.s004.pdf]
