# Supplementary material for: Meteorological Influences on the Incidence of Aneurysmal Subarachnoid Hemorrhage – A Single Center Study of 511 Patients
Source: PLoS One. 2013 Dec 2;8(12):e81621. doi: 10.1371/journal.pone.0081621 (PMC3847045; doi:10.1371/journal.pone.0081621)

Supplemental Figure S5

Histogram of the mean deviation from 2 days prior to the bleeding event from a Monte Carlo simulation (random selection of 511 days within the same time period) with 1000 re-samplings, assuming that no relationship between SAH and temperature exists. The green line corresponds to the observed mean given in Supplemental Figure 4 and the blue distribution results from the Monte Carlo simulation. The P-value gives the number of Monte Carlo member to the right of the observed value.

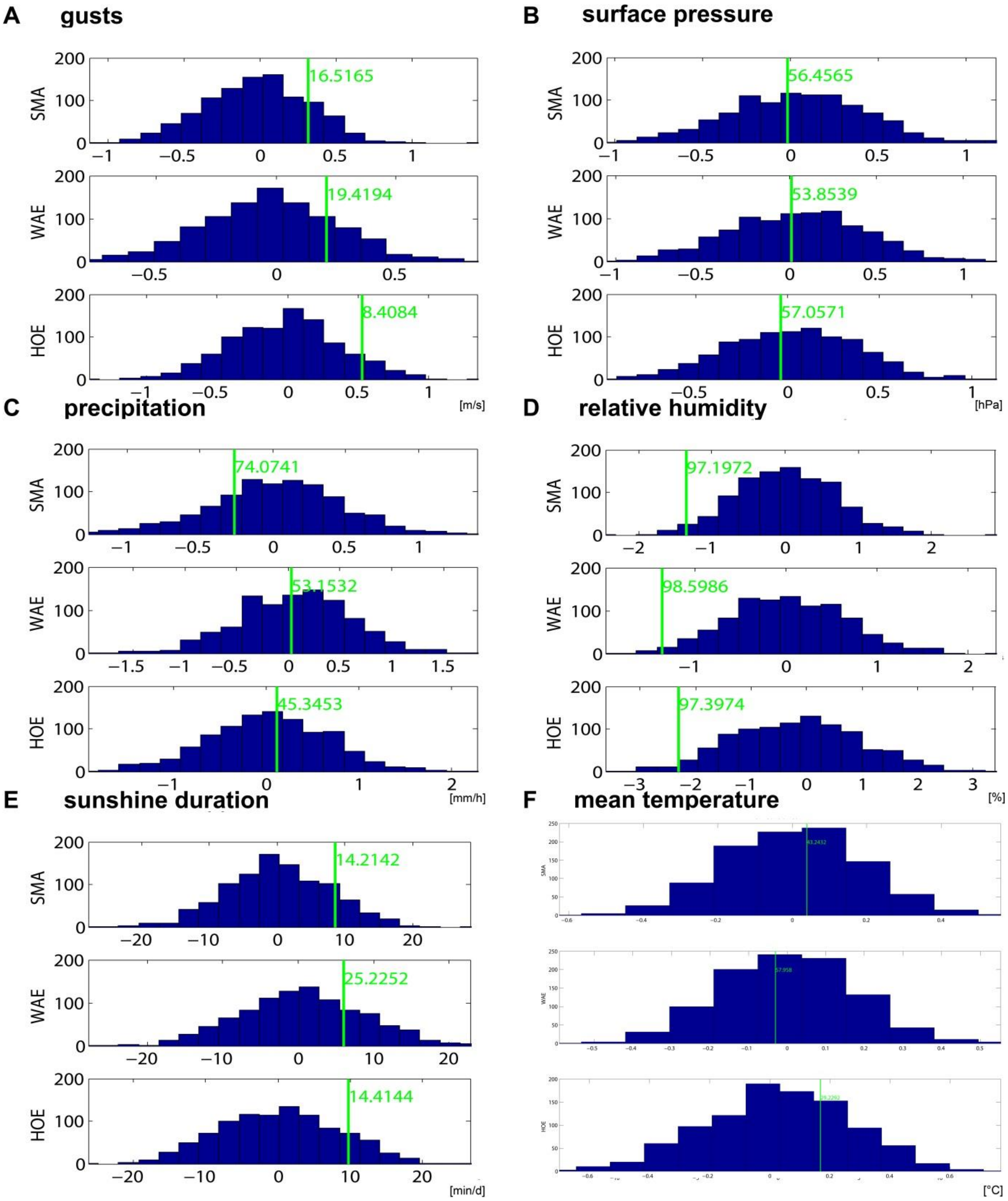

Supplement: Figure S5 — Histogram of the mean deviation from 2 days prior to the bleeding event from a Monte Carlo simulation (random selection of 511 days within the same time period) with 1000 re-samplings, assuming that no relationship between SAH and temperature exists. The green line corresponds to the observed mean given in Figure S4 and the blue distribution results from the Monte Carlo simulation. The P-value gives the number of Monte Carlo member to the right of the observed value. (PDF) [file pone.0081621.s005.pdf]
